# Today’s Public Health Issue: Workaholism

**Published:** 2017-02

**Authors:** Gülseren KESKİN

**Affiliations:** Atatürk Medical Technological Vocational Training School, Ege University, Bornova, İzmir, Turkey

## Dear Editor-in-Chief

Business addiction and workaholism concepts terms are used interchangeably. Oates firstly defines Workaholism as “Continually need to work in a manner that compulsive and uncontrollable” in the literature (1). This definition has come to the fore two issues: excessive work and an inner compulsion to work, uncontrolled manner. Workaholic’s most important difference from hardworking people are feeling extremely addictive to work, compulsive form of work, despite psychosomatic complaints continue to work, feel discomfort when they do not work they are willing to risk sacrificing work. Although many employees as workaholic people can also be an internal motivation to work, this job satisfaction shows a narcotic effect. In constant pursuit of workaholics to feel the effects of this work, it makes it an addiction (2).

However, there are eight characteristic symptoms of addictions in workaholism. First is the focus. These individuals are often seen as a treatment for study by themselves. Secondly mood variation; work on the basic regulatory function over individual feelings of the moment. Third tolerance; when it comes to work, there is a need for increasing the individual concerned. Fourth withdrawal symptoms; increased anxiety symptoms in the absence of the work. The fifth conflict; when encountering any problems in business life, they tend to enter into conflict with those around individuals. Sixth relapse, aggressive attitude towards work repeated in case of attacks. Eight ‘Reward dependence’ is a something that leads the way to become addicted for workaholic people (3, 4). Substances with addictive properties, especially ventral striatum, dopaminergic and opioids have powerful effects on the brain’s reward pathways involved in the system. The impact on the reward system often shows itself in the form of behavioral disorders. This forms the basis of the limbic system in the brain reward system is the basis for the perception of pleasure, and to control emotions and behavior such as to avoid dangerous or aversive situation, motivation condition. These symptoms are very similar to the withdrawal symptoms in the substance and behavioral dependency (5).

Behavioral addictions such as alcohol and substance abuse exhibit preoccupation, mood variability, tolerance, deprivation, interpersonal conflict and recurrence (relapse) that properties are the major components of dependence. Six criteria conforming behavior is defined as an “addiction” (6). Fundamental workaholism shows similar features, such as addiction (alcohol and drug or gambling addiction). Workaholism is not only an enthusiasm experienced here believed to be harmful negative consequences as well emotionally, socially and physically. Workaholic work obsessively beyond what is required by the job. When the work is impeded, the individual suffers destruction. This obsessional thought reveals a compulsive impulse control. Sometimes these compulsions may turn become destructive to individual; workaholics live conflict, memory, decision-making problem, sleep deprivation, negative or low mood. This situation can trigger depression (7).

The authors conclude that workaholism would appear an important topic of future work. Workaholism has not yet entered into any psychiatric diagnostic classification system. Workaholism is considered as a syndrome, a behavioral addiction, or a working attitude. Workaholism is discussed the current situation with the comprehensive psychological perspective in this study. A psychological evaluation in perspective etiology of workaholism and addiction relations that is important for preventing the negative results of disease and it would be evaluating as a form of dependence in terms of diagnosis and treatment in later years.
